# Investigating the effects of sleep and sleep loss on the different stages of episodic emotional memory: A narrative review and guide to the future

**DOI:** 10.3389/fnbeh.2022.910317

**Published:** 2022-08-29

**Authors:** Tony J. Cunningham, Robert Stickgold, Elizabeth A. Kensinger

**Affiliations:** ^1^Center for Sleep and Cognition, Department of Psychiatry, Beth Israel Deaconess Medical Center, Boston, MA, United States; ^2^Division of Sleep Medicine, Harvard Medical School, Boston, MA, United States; ^3^Department of Psychology and Neuroscience, Boston College, Chestnut Hill, MA, United States

**Keywords:** sleep, emotion, memory, consolidation, retrieval, encoding, reconsolidation, emotional memory

## Abstract

For two decades, sleep has been touted as one of the primary drivers for the encoding, consolidation, retention, and retrieval of episodic emotional memory. Recently, however, sleep’s role in emotional memory processing has received renewed scrutiny as meta-analyses and reviews have indicated that sleep may only contribute a small effect that hinges on the content or context of the learning and retrieval episodes. On the one hand, the strong perception of sleep’s importance in maintaining memory for emotional events may have been exacerbated by publication bias phenomena, such as the “winner’s curse” and “file drawer problem.” On the other hand, it is plausible that there are sets of circumstances that lead to consistent and reliable effects of sleep on emotional memory; these circumstances may depend on factors such as the placement and quality of sleep relative to the emotional experience, the content and context of the emotional experience, and the probes and strategies used to assess memory at retrieval. Here, we review the literature on how sleep (and sleep loss) influences each stage of emotional episodic memory. Specifically, we have separated previous work based on the placement of sleep and sleep loss in relation to the different stages of emotional memory processing: (1) prior to encoding, (2) immediately following encoding during early consolidation, (3) during extended consolidation, separated from initial learning, (4) just prior to retrieval, and (5) post-retrieval as memories may be restructured and reconsolidated. The goals of this review are three-fold: (1) examine phases of emotional memory that sleep may influence to a greater or lesser degree, (2) explicitly identify problematic overlaps in traditional sleep–wake study designs that are preventing the ability to better disentangle the potential role of sleep in the different stages of emotional memory processing, and (3) highlight areas for future research by identifying the stages of emotional memory processing in which the effect of sleep and sleep loss remains under-investigated. Here, we begin the task of better understanding the contexts and factors that influence the relationship between sleep and emotional memory processing and aim to be a valuable resource to facilitate hypothesis generation and promote important future research.

## Introduction

For nearly 100 years, investigators have researched sleep in the context of memory performance (Jenkins and Dallenbach, 1924; Ekstrand, 1967). Substantial evidence over this time has indicated that sleep before and after both learning and retrieval benefits multiple forms of declarative and non-declarative memories compared to matched periods of active wakefulness or sleep deprivation (Stickgold, 2005; Walker and Stickgold, 2006; Stickgold and Walker, 2007; Walker, 2008; Alger et al., 2014), though the exact mechanisms underlying this effect continue to be debated (see Nadel et al., 2000; Hobson and Pace-Schott, 2002; Ellenbogen et al., 2006; Squire et al., 2015; Yonelinas et al., 2019; Zadra and Stickgold, 2021). Around the turn of the millennium, an interest in the role of sleep in the preferential processing, retention, and retrieval of emotional episodic memories developed and continues on through today. Specifically, a series of notable studies indicated sleep, and perhaps rapid eye movement (REM) sleep in particular, retains or enhances memory for arousing or emotional information above and beyond neutral information of the same kind (e.g., words, pictures, etc.; Wagner, 2001; Hu et al., 2006; Holland and Lewis, 2007; Nishida et al., 2009).

More recently, however, a series of comprehensive meta-analyses and reviews exploring this preferential processing of emotional content during sleep, perhaps considered settled science by many, have called this effect into question (Lipinska et al., 2019; Schäfer et al., 2020; Davidson et al., 2021). With regard to the purported benefit that sleep during the early consolidation period has on the specific retention of emotional information, a recent sweeping review of the literature revealed that while a few studies do support this effect, the majority of studies *do not* support an interaction between sleep and emotion on long-term declarative memory processing (Davidson et al., 2021). In an effort to prevent the loss of the baby with the bathwater, rather than arguing against the role of sleep in emotional memory processing entirely, this review and others have noted a list of factors that need to be taken into consideration and controlled for in a stepwise manner in future research that will ultimately help to reveal the strength and limitations of this effect. These include factors such as the placement and quality of sleep in relation to the encoding, consolidation and retrieval of an emotional memory; the amount of time that has passed between encoding and retrieval; the content, context, and intensity of an emotional experience or stimuli; the types of probes and strategies used to assess memory at retrieval; whether there is pre- as well as post-sleep testing; the type of sleep manipulation employed (e.g., nap vs. full nights of sleep); and the macro- and microarchitecture of the studied sleep period (Lipinska et al., 2019; Schäfer et al., 2020; Davidson et al., 2021).

**FIGURE 1:**
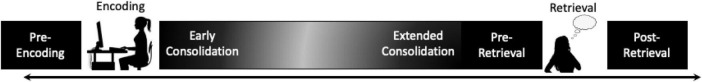
Schematic of the five stages of long-term episodic memory processing during which sleep can be manipulated: (1) prior to encoding, (2) immediately following encoding during early consolidation, (3) during extended consolidation away from initial learning, (4) just prior to retrieval of emotional memory, and (5) post-retrieval/reconsolidation.

The goal of the present review is to initiate this deeper exploration of the relationship between sleep and emotional memory processing by outlining the current state of the literature with regard to just one of the factors noted above: the *placement* of sleep (or sleep loss) in relation to the encoding, consolidation and retrieval of an emotional memory (i.e., when does the manipulated sleep period occur with relation to the different phases of memory processing). To do so, we have identified and defined five different stages during which sleep can be manipulated that may have overlapping or distinct effects on the formation, evolution, and retrieval of an emotional memory (see Box 1 and Figure 1):

**BOX 1. Operationalized definitions for each phase of emotional memory processing that may be influenced by sleep.**

**Table:** 


Pre-encoding	The period prior to the encoding of emotional information or the experience of an emotional event.
Early consolidation	The period just following encoding of an emotional event, during which memories are thought to be highly labile and susceptible to additional modulation via factors such as sleep and stress. The precise amount of time that this period lasts before it enters into the next phase (extended consolidation) remains an open question, especially with regard to emotional memories.
Extended consolidation	Any additional consolidation processing that occurs to a memory after it transitions from its labile state during the early consolidation phase into a more stable memory trace. This phase of memory processing extends all the way until the memory is retrieved, reactivated, or forgotten.
Pre-retrieval	The period just prior to the activation of a memory trace in response to a cue to call to mind a specific item or event or to perform a specific task (i.e., the period just prior to memory testing)
Post-retrieval/reconsolidation	Immediately after a memory is retrieved or reactivated it enters the reconsolidation phase of memory processing, during which it returns to a labile state that again makes it more prone to influence, modulation, and forgetting, much like the period of early consolidation. This phase can lead to updates to an already-existing memory trace or can lead to the creation of an additional memory trace that may interfere with the originally created trace. Similar to the early consolidation period, the exact amount of time that a memory spends in this labile phase post-retrieval remains an open investigation.

(1) Prior to encoding emotional information^1^,

(2) Immediately following encoding during early consolidation,

(3) During extended consolidation away from initial learning,

(4) Just prior to retrieval of emotional memory^2^, and

(5) Post-retrieval as an emotional memory may be restructured and reconsolidated.

We briefly review some of the previous literature that falls primarily under each category with the hope of shedding light on how sleep influences may differ in strength across these five stages. Perhaps more importantly, however, the additional objectives of this review are to (1) identify problematic overlaps between memory stages in commonly used sleep and emotional memory protocols and make suggestions to prioritize certain study designs in the future that will provide the opportunity to better disentangle the distinct role of sleep at each phase of emotional memory processing^3^ (see Box 2 for a demonstration), and (2) highlight areas for future research by identifying the stages of emotional memory processing that remain under-investigated with respect to their relationship with sleep.

BOX 2. Demonstration of the issue*.

Je’Rell is a budding graduate student interested in how sleep loss affects emotional memory consolidation. In his first study, Je’Rell had participants come into the sleep lab at 9pm. They were then either allowed a normal night of sleep or were totally sleep deprived. In the morning, Je’Rell had participants view negative and neutral scenes, and an hour later they completed a recognition memory test for the scenes. Je’Rell found that not only did sleep deprived participants have poorer memory overall, but that memory for both scene types deteriorated equally. Participants in the sleep condition, however, showed greater memory for the negative scenes compared to the neutral scenes. From this, Je’Rell concluded that sleep *prior to encoding* allows for the preferential memory processing of emotional over neutral information.

Excited, Je’Rell turned to his lab mate Hachirô to tell him the news but was only met with muted enthusiasm. Hachirô told Je’Rell that he too had just finished his study on sleep and emotional memory. In his study, Hachirô also had his participants come into the sleep lab at 9pm. However, Hachirô had his participants do the encoding of the negative and neutral scenes shortly after arriving that evening. Immediately following encoding, Hachirô’s participants were either allowed a normal night of sleep or were totally sleep deprived. The next morning, participants also completed a recognition memory for the scenes. Just like Je’Rell, Hachirô found that participants in the sleep deprived group had poorer memory overall and that memory for both scene types deteriorated at the same rate, while those in the sleep condition showed better overall memory and a preference in memory for negative over neutral scenes. From this, Hachirô concluded that it was sleep during the *early consolidation* phase that was the critical process for the enhancement of emotional over neutral memories.

After heated debate over a number of pints and Diet Cokes, Je’Rell and Hachirô finally turned to the lab postdoc, Luisa, to settle the debate. Before they could, Luisa excitedly told them about her recent findings. In her study, Luisa had all of her participants view negative and neutral scenes at 9pm, followed by a normal night of sleep. Seventy-two hours later, Luisa had her participants return to the sleep lab and half of the participants were allowed a normal night of sleep while the other half were sleep deprived for the night. In the morning, the participants completed a recognition memory test for the scenes, and you guessed it. Once again, sleep deprived participants showed poorer retention for memory overall with both types of memory deteriorating at the same rate, while those that slept showed superior memory overall and enhanced memory for negative compared to neutral scenes. From this, Luisa concluded that sleep *prior to retrieval* was critical for the enhancement of emotional over neutral memories. The silence is deafening as the three friends look at each other. Who is right?

We would argue that across these scenarios, while none of the interpretations can be definitively determined to be incorrect, the study design used by Luisa allows us to be the most confident in her results, as she did the best job isolating the phase of memory that she was targeting (sleep and sleep loss prior to retrieval). In contrast, multiple phases of memory processing were affected in the study designs employed by Je’Rell (encoding, consolidation, retrieval) and Hachirô (consolidation, retrieval). While all three studies are helpful in developing our understanding of the contexts and circumstances that sleep and sleep loss can affect emotional memory performance, only Luisa’s was designed in such a way that allows substantial confidence in the specific *stage* of emotional memory processing that was directly influenced.

*Note the results here are hypothetical to help illustrate our point and may not reflect the majority of the current state of the literature.

Of note, while the primary focus of this review is emotional episodic memory processing in humans, some of the identified stages of memory processing remain nearly or entirely unexplored in the context of human sleep. In these instances, examples from the animal literature are used to provide readers with the extent of our current knowledge and in the hopes of inspiring additional research. It is important to acknowledge, however, that emotional episodic memory in humans and animal models of “emotional memory” – typically fear conditioning – may be vastly different in both expression and underlying mechanisms. We also limited the scope of our review here specifically to the effect of sleep at each stage of long-term episodic memory processing for emotional vs. neutral information. This is not because we think that the placement of sleep is disproportionately important for emotional episodic memories: Substantial evidence suggests that other forms of declarative and non-declarative memories may also be affected by when sleep is present (Stickgold et al., 2000; Fischer et al., 2002; Walker et al., 2002, 2003; Stickgold, 2005; Walker and Stickgold, 2005). However, the role of sleep in emotional memory processing may have particular clinical relevance. Remembering negative events that we experience can be critically important to our ability to make adaptive decisions and even to survive, but it can be detrimental to re-experience too much emotion and stress each time these past events are recalled. It has been hypothesized that sleep helps to protect or boost memory for the content of an emotional experience while simultaneously allowing for or assisting with the reduction in emotional tone associated with the event (Walker and van Der Helm, 2009; Goldstein and Walker, 2014). As such, it has been suggested that sleep not only plays a critical part in the healthy processing of our day-to-day emotional experiences, but also of traumatic experiences as well (Walker and van Der Helm, 2009; Tempesta et al., 2010; van der Helm et al., 2011; Motomura et al., 2013; Stickgold and Walker, 2013; Cunningham et al., 2014a; Goldstein and Walker, 2014; Thormar et al., 2014; Harrington and Cairney, 2021; Zeng et al., 2021). Regardless of the severity of the emotional experience, this adaptive processing is thought to be highly beneficial for our overall well-being and mental health (Walker and van Der Helm, 2009; Cunningham et al., 2014b; Cunningham and Payne, 2017). This theory is in striking contrast to a separate line of research that has suggested depriving sleep following a traumatic experience with the goal of preventing the onset of PTSD symptoms (Kuriyama et al., 2010; Cohen et al., 2017). The juxtaposition between these competing theories is apparent and of potentially grave importance. Thus, while our goal here is to initially promote future research aimed at taking a dedicated, stepwise approach to determining how sleep affects each phase of emotional episodic memory processing, ultimately we believe this treatment should be extended to all forms of human memory consolidation.

### Process S vs. Process C

**FIGURE 2:**
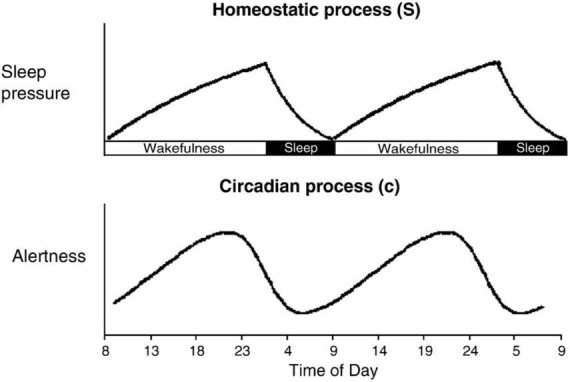
Process S is a sleep-dependent process that reflects the homeostatic sleep pressure that builds up linearly as long as we are awake (i.e., the extent of time that has passed since our previous bout of sleep). Process C is a sleep-independent process that reflects our internal daily rhythms kept in sync with the Earth’s near 24-h cycle through the suprachiasmatic nucleus (SCN). Adapted from Stiller and Postolache (2005).

When considering the placement of sleep and wakefulness, it is important to note that more than just the order and proximity of the next sleep episode needs to be taken into consideration, and this may be particularly true when emotional processing is involved. Two critical components that are inextricably embedded into every sleep and memory study are Process S and Process C (see Figure 2; Borbély and Achermann, 1999; Carskadon et al., 2004; Stiller and Postolache, 2005; Borbély, 2009). Process S (or Process H) refers to the homeostatic sleep pressure that builds up over time spent awake. From the moment we rise in the morning, Process S begins to accumulate until our next bout of sleep. This sleep-dependent process reflects the growing need to recover from the wear and tear of wakefulness through the buildup of sleep-promoting hormones, like adenosine, driving the need for sleep, and especially slow wave activity (SWA) during sleep, to restore optimal functioning (Carskadon et al., 2004; Landolt, 2008). Quite simply, the longer you are awake, the more your brain craves sleep.

Working in tandem with Process S is Process C. Process C is our internal body clock that is kept in sync with the Earth’s ∼24-h cycle primarily via light exposure through the suprachiasmatic nucleus (SCN; Czeisler and Klerman, 1999; Carskadon et al., 2004; Mistlberger, 2005). Process C can favor sleep or wakefulness at different times of day depending on our own internal daily oscillations. In addition to promoting sleep and wakefulness, Process C exerts influence on a host of different endogenous physiological functions, including body temperature, metabolism, immune system function, and melatonin, cortisol, and other hormone production, just to name a few (Czeisler and Klerman, 1999; Carskadon et al., 2004). As demonstrated in Figure 2, under normal circumstances with a typically entrained circadian rhythm, Process C has a vastly different influence at 8am as compared to 8pm.

With regard to exploring the role of sleep in emotional memory processing, taking Process S and Process C into careful consideration may be even more critical than when investigating other forms of memory. This is because both extended periods of wakefulness (i.e., sleep deprivation) and different periods in our circadian rhythm have both been shown to affect cognitive function (Dijk et al., 1992; Drummond et al., 2004; Durmer and Dinges, 2005; Blatter and Cajochen, 2007; Goel et al., 2009; Killgore, 2010; Lim and Dinges, 2010; Krause et al., 2017) and emotional perception (Paradee et al., 2008; Walker and van Der Helm, 2009; Kahn et al., 2013; McClung, 2013; Prather et al., 2013; Goldstein and Walker, 2014; Stolarski and Jankowski, 2015). If the expectation is that our emotion and memory systems interact in such a way that drives preferential long-term declarative memory for arousing over neutral information (van der Helm and Walker, 2011; Cunningham et al., 2014b; Cunningham and Payne, 2017) and both Process S and Process C individually exert influence on these systems, then in order to understand the independent effects of sleep on emotional memory processing, Process S and Process C must both be taken into consideration.

While Process S and Process C cannot be completely controlled in many study designs, there are some elements or groups that can be added to most study protocols that can help. One of these additions is to conduct an initial memory test after just a short delay, immediately following encoding^4^. Not only does comparing pre-sleep to post-sleep memory performance better isolate the effects of sleep manipulations that occur following encoding (e.g., during early consolidation), but potential “time of day” effects can be determined and controlled for statistically. For instance, if encoding takes place at different times of day (e.g., 9am and 9pm) then the homeostatic pressure (Process S) by definition is going to be substantially different between groups as the 9pm group has 12 additional hours of sleep pressure built up compared to the 9am group. If this immediate test reflects no initial performance differences between groups, it provides some support that Process S is not substantially altering the encoding of the material (though does not eliminate the possibility entirely), and if there is a difference, then it can be added to the analysis as a covariate.

Use of circadian control groups is another technique that can be implemented to help to account for the influences of Process S and Process C, especially if investigators have concerns that immediate testing may influence the long-term memory performance they plan to test (see *Footnote 4*). As described above, if encoding and retrieval are conducted at different times of day (e.g., wake: encoding 9am, retrieval 9pm vs. sleep: encoding 9pm, retrieval 9am) then in addition to the presence and absence of sleep between sessions, the innate circadian rhythms of the groups are exerting very different physiological influences at the time of encoding, initial consolidation, and retrieval. Circadian control groups can be recruited to complete emotional memory encoding and retrieval tasks after a shorter delay (e.g., 30–60 min) such that the entire protocol can be conducted in a single session. Again, while it does not completely eliminate the possibility that circadian influences might account for some of the effect, if there is no difference between the circadian control groups then it does provide support that the circadian influences during encoding, early consolidation, and retrieval do not completely account for the differences found between the sleep and wake conditions, and if there are differences they can potentially be controlled for statistically. Moreover, collaborations between sleep and circadian researchers could lead to the implementation of even more intensive, elegant study designs that disentangle the effects of Process S from Process C at the neurophysiological level (see Discussion section).

A final biological rhythm that may be particularly important in the discussion of emotional memory processing is the regulation of rapid eye movement (REM) sleep. While the need and intensity for slow wave activity (SWA) during non-REM (NREM) seems to largely increase linearly over time spent awake (Dijk et al., 1990), REM sleep seems to have its own regulatory mechanisms. For one, depriving REM sleep, as might happen during a sleep-restriction protocol in which participants are only allowed to sleep the first half of the night, leads to an immediate REM sleep rebound and reduction in the intensity of NREM sleep in the subsequent sleep periods (Beersma et al., 1990; Brunner et al., 1990). Unlike SWA, however, propensity for REM sleep has also been shown to be closely coupled to circadian rhythm (Czeisler et al., 1980), and the relationship between REM sleep and learning has been shown to be affected by the circadian phase during which the REM sleep occurs (Cajochen et al., 2004). Importantly, it has been hypothesized that REM sleep may play a particularly important role in the processing of emotional memories (Walker and van Der Helm, 2009; van der Helm and Walker, 2011; Cunningham et al., 2014b), yet – to our knowledge – the timing of its effectiveness with regard to circadian phase has never been investigated in the context of emotional memory. Thus, relative to other stages of sleep, the distinct and complex regulation of REM sleep may be particularly important in the investigation of sleep’s effect in emotional memory. As we now move into discussion of the current state of the field and dissect the literature based on the placement of the sleep manipulation with regard to emotional memory processing, we encourage innovative thought as to how some of these REM-related factors may be better accounted for in future research.

## Sleep placement and emotional memory processing

### Sleep prior to encoding

Sleep can be manipulated in the period prior to the encoding of emotional information or the experience of an emotional event. This includes both the presence or absence of sleep (e.g., sleep vs. sleep deprivation or nap vs. no nap designs), as well as the duration of time since the previous bout of sleep leading to differences in the magnitude of homeostatic sleep pressure at encoding (e.g., nocturnal sleep vs. daytime wakefulness designs).

Sleep prior to emotional experiences may be particularly important for the initial experience of emotionality that shapes the development of the subsequent memory, as the absence of sleep has been shown to heavily affect our mood (Carskadon and Dement, 1979; Reddy et al., 2017; Feng et al., 2018; Ben Simon et al., 2020; Tomaso et al., 2021), alter our perception of and response to emotional information (van der Helm et al., 2010; Minkel et al., 2011; Alfarra et al., 2015; Killgore et al., 2017; Tomaso et al., 2021), and inhibit our ability to regulate our emotional response when perceiving or experiencing emotional events (Killgore et al., 2008a; Killgore, 2013; Baum et al., 2014; Miller et al., 2015; Tomaso et al., 2021). Intriguingly, a growing body of evidence has indicated that sleep loss prior to viewing emotional stimuli may lead to hyperactivity of limbic regions such as the amygdala (Yoo et al., 2007a; Goldstein et al., 2013; Motomura et al., 2013; Goldstein-Piekarski et al., 2015), altered amygdala-prefrontal cortex (PFC) connectivity (Chuah et al., 2010; Killgore, 2013; Motomura et al., 2013), impaired or blunted behavioral responses to aversive stimuli (van der Helm et al., 2010; Alfarra et al., 2015; Pilcher et al., 2015; Killgore et al., 2017), and increased emotional responses to neutral stimuli (Tempesta et al., 2010; Simon et al., 2015). It has been suggested that these features may lead the sleep-deprived brain to treat all stimuli as potentially aversive, possibly due to a decreased threshold for emotional activation (Goldstein et al., 2013; Simon et al., 2015), which would likely have an impact on the subsequent memory processing for both emotional and neutral stimuli. It is also worth noting that animal models have indicated that substantial sleep loss in rodents prior to operant conditioning leads to impaired contextual memory (but not cued memory; McDermott et al., 2003). The authors further provide evidence that this may be due to cellular-level alterations in membrane excitability leading to reduced hippocampal long-term potentiation (LTP), thus impairing hippocampal-dependent learning performance.

With regard to declarative memory in humans, studies using neutral stimuli only have consistently shown an impairment in subsequent memory when sleep is deprived or impaired prior to encoding (Drummond et al., 2000; Yoo et al., 2007b; Chuah et al., 2009; Van Der Werf et al., 2009; Saletin et al., 2016), though certain structural brain morphology (Saletin et al., 2016) and compensatory activation (Drummond et al., 2000; Chuah et al., 2009) may limit the negative effects that sleep loss at encoding has on memory performance. Similar detrimental effects have been reported in studies testing emotional declarative memory (see Table 1). In one study, participants were sleep deprived for 36 h prior to encoding negative, neutral, and positive words, and then were allowed two nights of recovery sleep before testing (Walker and Stickgold, 2006). They found that sleep-deprived participants had a 40% reduction in overall memory with the magnitude of the deficit differing across emotional categories. Specifically, retention was especially poor for neutral and positive words, with the retention in memory for positive words reaching a 59% deficit, while negative words had a smaller, non-significant decrease (19%). Another study either allowed normal sleep or totally sleep-deprived participants prior to viewing negative, neutral, and positive videos (Tempesta et al., 2016). With total sleep deprivation (TSD), recognition memory for video images was once again impaired for positive and neutral scenes, while recognition for negative scenes was similar between rested and TSD participants. With regard to contextual memory (i.e., temporal order of scenes), TSD impaired performance overall, independent of emotional valence (Tempesta et al., 2016).

**TABLE 1:** Studies aimed at investigating the effect of sleep on the pre-encoding phase of emotional memory processing.

Study	Human subjects (Y/N)	Genders	Age (M ± SD)	N	Between-groups/Within-groups	Sleep manipulation	Stimulus material	Emotions	Immediate test (Y/N)	Time of day control (Y/N)	Main effect of sleep manipulation (effect size)	Interaction sleep × emotion (effect size)
Cellini et al., 2016	Y	Mixed	23.81 ± 2.63 years	46	Between	Nap	Pictures	NNP	N	N	Y (η2 = 0.15)	N (NR)
Kaida et al., 2015	Y	Men	21.4 ± 1.65 years	14	Within	TSD/S	Pictures	NNP	N	N	Y (ε = 1.00)	N (NR)
Kaida et al., 2015	Y	Men	22.0 ± 2.09 years	18	Within	REMD/S	Pictures	NNP	N	N	N (NR)	N (NR)
Tempesta et al., 2016	Y	Mixed	21.6 ± 2.1 years	48	Between	TSD/S	Videos	NNP	N	N	Y (NR)	Y (NR)
Walker and Stickgold, 2006	Y	NR	NR	NR	Between	TSD/S	Words	NNP	N	N	Y (NR)	NR

Style of Tables adapted from Davidson et al. (2021). Table includes studies included as part of this narrative review, and is not necessarily a comprehensive list of all published works. Immediate test, test prior to sleep manipulation; Y, yes; N, no, Mixed, not restricted to a single reported gender; DW/NS, daytime wake vs. nocturnal sleep; TSD/S, total sleep deprivation vs. sleep; REMD/S, REM sleep deprivation vs. sleep; Nap, daytime nap vs. no nap protocol; NNP, negative, neutral, positive stimuli; NR, not reported.

Kaida et al. (2015) had participants encode positive, neutral, and negative scenes following TSD, targeted rapid eye movement sleep deprivation (REMD), or normal sleep. Following the sleep manipulation, participants completed a recognition task for half of the previously viewed material^5^, and returned 1 week later to complete an identical recognition task for the other half of the material. TSD led to impaired overall memory performance, independent of emotional valence; while the decrease between testing sessions appeared more pronounced in the TSD condition, the Condition × Day interaction did not reach significance. Compared to the control condition, REMD had no significant impact on overall picture recognition, although an exploratory analysis of just negative pictures found that REMD participants remembered slightly more negative pictures on Day 1 and slightly fewer negative pictures on Day 8, leading to a significant Condition × Day interaction for negative picture memory (Kaida et al., 2015). A nap study manipulated sleep both prior to encoding and during early consolidation by having participants encode two sets of pleasant, unpleasant, and neutral images, one prior to nap or wake and another after nap or wake, followed by a memory test for all images 30 min after encoding the second set (Cellini et al., 2016). Participants that napped had better overall memory for both sets of images, indicating that sleep both pre- and post-encoding facilitates learning. While the images encoded pre-sleep showed an effect of valence (neutral and unpleasant stimuli were better discriminated than pleasant stimuli), the effect of valence did not reach significance for the images that were encoded immediately following sleep (Cellini et al., 2016).

**FIGURE 3:**
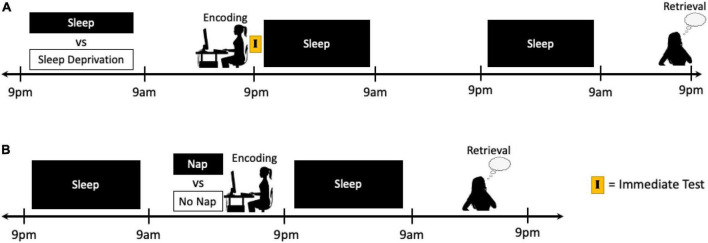
Schematics of two potential study protocols designed to target the effects of sleep manipulation on the pre-encoding period of emotional memory processing. **(A)** Following a night of sleep or sleep deprivation, participants can complete an encoding task just prior to the next night of sleep, thus manipulating sleep prior to encoding while allowing both groups to sleep during the early consolidation phase and be rested again prior to final retrieval. **(B)** Typically rested participants can remain awake or be given a nap opportunity prior to an encoding task, and then all participants can be given a normal night of sleep prior to testing the next day. The primary challenge in targeting the pre-encoding phase of memory processing is distinguishing the specific effects of the sleep manipulation on the encoding of information from any lingering effects that the sleep manipulation may have during the early consolidation period.

The pre-encoding phase in emotional memory processing can begin to be targeted in study designs by manipulating sleep prior to the encoding of emotional information (e.g., sleep vs. sleep deprivation) and allowing normal periods of sleep to occur immediately following encoding and before testing (Figure 3A). Given that the early consolidation phase begins *immediately* after the cessation of the encoding task, disentangling the effects of sleep prior to encoding from lingering effects of the sleep manipulation during the early consolidation phase may be one of the most challenging distinctions to make. Moreover, the micro- and macro-architecture of the sleep period immediately following encoding would also need to be taken into consideration. Polysomnography (PSG) sleep recordings (particularly with high-density capabilities; Cox et al., 2012, 2017, 2018a; Cox and Fell, 2020; Cunningham et al., 2021a; Denis et al., 2021) and an immediate test directly following encoding may help to at least partially account for some of these influences. Nap studies could also be conducted that either permit or deny a nap period prior to encoding, and then delay testing until subsequent nocturnal sleep is obtained in both groups (Figure 3B). In study designs that are unable to keep the encoding and testing sessions consistent between conditions, differences in circadian rhythm, time of day effects, and duration of time since the previous sleep episode may be controlled for using immediate testing or circadian control groups, as described above.

### Sleep during early consolidation

The early consolidation period just following encoding of an emotional event is a period during which memories are thought to be highly labile and susceptible to additional modulation via factors such as sleep and stress (McGaugh, 2000, 2004, 2018; Dudai et al., 2015; Squire et al., 2015). Withholding sleep (i.e., sleep deprivation) during this critical period has been shown to prevent long term memory storage for some types of memories (Stickgold et al., 2000; Stickgold, 2005; Walker and Stickgold, 2006). Likely because seminal models of emotional memory focused on the role of emotional arousal in modulating consolidation processes (McGaugh, 2000, 2004), the vast majority of sleep and emotional memory research has focused on the manipulation of sleep during this critical period with early evidence suggesting that the sleeping brain is able to specifically target emotional content for preferential memory processing (Wagner, 2001; Hu et al., 2006; Holland and Lewis, 2007; Nishida et al., 2009).

Recent reviews and meta-analyses have extensively reviewed the literature focusing on this stage of emotional memory consolidation and highlighted other factors that may influence the effect of sleep during this period (see Lipinska et al., 2019; Schäfer et al., 2020; Davidson et al., 2021; for review). The quick synopsis, however, is that while several studies do report an interaction between sleep and emotion during the early consolidation period (Hu et al., 2006; Wagner et al., 2006; Nishida et al., 2009; Prehn-Kristensen et al., 2009, 2013; McKeon et al., 2012; Chambers and Payne, 2014; van Heugten-van der Kloet et al., 2015; Bolinger et al., 2018; Cox et al., 2018b), most do not (e.g., Wagner et al., 2007; Baran et al., 2012; Morgenthaler et al., 2014; Ackermann et al., 2015, 2019; Göder et al., 2015; Tempesta et al., 2015, 2017; Gilson et al., 2016; Jones et al., 2016, 2018; Lehmann et al., 2016b; Harrington et al., 2018; Ashton et al., 2019; Bolinger et al., 2019; Huguet et al., 2019; Kurz et al., 2019; Schoch et al., 2019; Cross et al., 2020; Huan et al., 2020; for Tables detailing the current state of the literature on the effect of sleep during the early consolidation stage of emotional memory processing see recent open access publication Davidson et al., 2021). A potential confound in a number of these studies, however, is that sleep is frequently manipulated at multiple stages of memory processing, such as during early consolidation *and* prior to retrieval. Recent models of emotional memory have highlighted that the effects of emotion on memory are not constrained to the consolidation phase, with emotion robustly affecting retrieval processes (e.g., Bowen et al., 2018; Talmi et al., 2019). Thus, manipulations that simultaneously target both consolidation and retrieval may be blending effects that could plausibly be working in opposition to one another. As such, two studies in particular are worth noting as their study designs allowed for above average isolation of the sleep manipulation during the early consolidation period.

Tempesta et al. (2017) had participants view six films (two negative, two positive, two neutral) before either TSD or a normal night of sleep. They were then allowed multiple nights of recovery sleep before returning to the lab 48 h later for a surprise recognition test of scenes from the videos. They found that the sleep deprived participants had poorer overall memory, regardless of film valence. Similarly, Wagner et al. (2007) had participants view happy, angry, and neutral faces prior to a night of TSD or normal sleep in a within-subject, crossover design. They were then allowed a night of recovery sleep before returning for the memory test. They too found that while sleep deprivation impaired overall memory accuracy, there was no interaction with stimuli valence.

The one exception to this growing trend of null results are studies that utilize emotional trade-off paradigms as the assessment for memory (Davidson et al., 2021). In these study designs, images are created that display either a negative or neutral central object overlayed over a neutral background (Kensinger et al., 2007; Cunningham et al., 2014a,c, 2021b). During encoding, participants view the entire scene (e.g., a spider on a fence), but at recognition the scene components are separated and presented one at a time (e.g., participants see just the spider and just the fence on different trials). Unlike studies using other types of emotional memory probes, a majority of these studies have demonstrated an interaction with sleep and emotional memory, such that the magnitude of the difference between negative objects and their paired backgrounds is larger after sleep than after similar periods of wakefulness (e.g., Payne et al., 2008, 2012, 2015; Payne and Kensinger, 2011; Alger et al., 2018). What is remembered about these types of complex emotional scenes has also been shown to be affected by the way memories must be retrieved (Madan et al., 2020), suggesting the possibility that sleep’s exaggeration of emotional memory trade-offs may be reflecting effects on both consolidation and retrieval. However, in at least one study using a 24-h delay (Payne et al., 2012), the effect of sleep on the emotional memory trade-off was greatest when sleep occurred soon after learning (disproportionately affecting early consolidation) rather than when it was shifted in time to occur during extended consolidation, just before retrieval. Interestingly, while the pattern of these results was similar for both a strict, specific assessment of memory performance and a more lenient, general memory performance measure, the strength of the effect was stronger in the stricter measure of veridical memory (Payne et al., 2012). Other studies that probe different aspects of emotional memory or different strengths of the emotional memory trace (e.g., specific vs. gist memory, direct vs. associative memory, “Remember” vs. “Know,” etc.) have also demonstrated differences in the magnitude or even direction of the sleep effect depending on which aspect of memory performance was assessed (e.g., Hu et al., 2006; McKeon et al., 2012; Goldschmied et al., 2015; Alger and Payne, 2016; Schoch et al., 2017; Kurz et al., 2019). Thus, the type of probe or the inclusion of false alarm rates in the calculation of memory performance (e.g., hit rate vs. d-prime) may interact with the placement of sleep and is in need of careful consideration to add precision to our understanding of sleep’s relationship with emotional memory processing (see section “Discussion”).

In summary, recent reviews and meta-analyses have indicated that the specific memory enhancement of emotional over neutral information due to sleep during the early consolidation stage may not be as strong as initially indicated (i.e., sleep may more frequently benefit both emotional and neutral memories), and may depend on a variety of additional factors, such as the type of emotional stimuli used, the use of pre- and post-sleep testing to determine changes from baseline memory, and the type of memory probe, such as free recall vs. recognition testing (Lipinska et al., 2019; Schäfer et al., 2020; Davidson et al., 2021). This phase of memory processing may also be subject to time of day and circadian influences as hormones and emotional reactivity fluctuate significantly throughout the day. Moreover, additional research is needed to determine the exact duration of this “critical labile period” where memories can be more easily influenced compared to the extended consolidation period (see below) at which time memories may be less influenced to modulation unless reactivated. Recent research exploring forgetting curves have indicated that this labile period may last up to 12 h or more in the absence of sleep for some types of memory (Radvansky et al., 2022), and this may be subject to individual differences as well.

**FIGURE 4:**
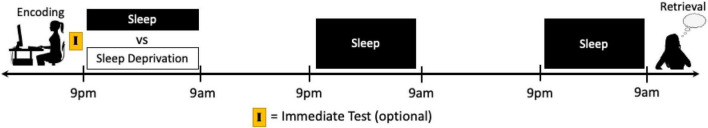
Schematics of a potential study protocol designed to target the effects of sleep manipulation on the early consolidation stage of emotional memory processing. Participants can complete an encoding task immediately prior to a period of sleep or sleep deprivation. By allowing multiple nights of recovery sleep prior to retrieval, the effects of the sleep manipulation can be limited primarily to the early consolidation period. Moreover, an immediate test directly after encoding can be used to determine within-subject memory changes due to the different conditions. However, it must be kept in mind that the testing of memory itself may influence subsequent memory performance (see Footnote 4 and section “Discussion”).

Critically, a majority of studies that have sought to explore the role of sleep during early consolidation on the long-term formation of emotional memories have inadvertently manipulated sleep prior to retrieval as well. For example, in many nap vs. no nap and nocturnal sleep vs. daytime wakefulness protocols, the buildup of sleep homeostatic pressure at retrieval also differs between groups. Similarly, in sleep vs. sleep deprivation studies, sleep at retrieval is directly influenced unless recovery sleep is obtained. As such, the impact of sleep during early consolidation can be isolated by manipulating sleep immediately following exposure to emotional information (e.g., nap vs. no nap design, sleep vs. sleep deprivation design) and then allowing all participants to obtain subsequent night(s) of recovery sleep prior to testing (Figure 4). These designs would also control for circadian rhythm, time of day effects, and duration of time since previous sleep episodes by keeping the encoding and retrieval sessions consistent between groups.

Another intriguing line of research that has thus far primarily been implemented during the early consolidation phase of memory processing is the use of non-invasive stimulation techniques during sleep, such as targeted memory reactivation (TMR; Oudiette and Paller, 2013), closed- and open-loop auditory stimulation (Weigenand et al., 2016; Baxter et al., 2020), and transcranial direct current stimulation (tDCS; Marshall et al., 2004; Zhang and Gruber, 2019). Generally, the goal of these techniques is to affect or enhance sleep in such a way that it will ultimately boost its effect on both emotional and non-emotional types of memory. While a complete review of studies utilizing these techniques is beyond the scope of the current review, TMR and other non-invasive stimulation techniques could be similarly employed at various stages of emotional memory processing and may be a valuable tool for increasing the precision of our understanding of sleep’s effect on different stages of memory processing (see Box 3 for additional discussion).

**BOX 3. Targeted memory reactivation (TMR) and non-invasive stimulation.**

A valuable addition to the conversation of creative study design is recent innovations in the modulation of the sleep periods. Determining performance changes following the enhancement or suppression of certain elements of sleep microarchitecture (e.g., slow waves, spindles, REM density, etc.) or by otherwise affecting the processing occurring during the sleep period may be another way to not only help us distinguish the impact of sleep on emotional memory processing, but also may help to reveal what elements of sleep play a primary role. As noted, the primary goal of some of these recent strategies — such as closed/open-loop auditory stimulation (Weigenand et al., 2016; Baxter et al., 2020) and transcranial direct current stimulation (tDCS; Marshall et al., 2004; Zhang and Gruber, 2019) — is simply to affect the sleep architecture to enhance or suppress certain elements of sleep. As we make suggestions for different study designs geared toward isolating the effects of sleep on the different stages of emotional processing, it is certainly worth consideration of how these techniques might also be implemented within each protocol to further our understanding of sleep’s effect.

Another relatively novel technique, Targeted Memory Reactivation (TMR), may be particularly relevant in the context of the present review. In TMR protocols, stimuli during encoding are typically either presented audibly or are paired with an auditory or olfactory cue. Then, *during* the subsequent sleep period, previously experienced auditory stimuli or paired cues may or may not be presented again along with novel foils at a level that is designed to be processed by the brain without waking the participant. The goal of this stimulation is to artificially trigger the memory engram for replay during the sleep period. In this way, TMR has elements of encoding, consolidation, and reconsolidation all occurring within a sleep period, and in particular may be a way to enhance the consolidation or expedite the reconsolidation phase of emotional memory processing. TMR has already generated some very interesting findings in the overall sleep and memory field (see Oudiette and Paller, 2013; Schouten et al., 2017; Cellini and Capuozzo, 2018) and we are beginning to understand more about the underlying mechanisms that promote memory enhancement through TMR (Lewis and Bendor, 2019). TMR has been shown to reliably enhance declarative memory (e.g., Rasch et al., 2007; Diekelmann et al., 2011; Donohue and Spencer, 2011; Van Dongen et al., 2012), procedural memory (e.g., Antony et al., 2012; Cousins et al., 2014; Schönauer et al., 2014), and creative performance (Ritter et al., 2012), with a recent meta-analysis indicating that TMR is highly effective, particularly when implemented during NREM sleep (Hu et al., 2020).

TMR has also been implemented at an encouraging rate with emotional stimuli, though the findings remain unsurprisingly mixed. Studies using fear extinction memory in humans have found that TMR may facilitate fear extinction learning (Hauner et al., 2013; He et al., 2015). Intriguingly, some animal literature suggests that TMR may also weaken or interfere with extinction learning (Rolls et al., 2013). With regard to emotional picture recognition, TMR has been shown to both benefit (Cairney et al., 2014; Lehmann et al., 2016a; Groch et al., 2017) and have no effect on (Ashton et al., 2018) subsequent memory performance. One final study found that TMR had no effect on the emotional memory trace, but did influence the affective tone (Rihm and Rasch, 2015). Overall, substantial evidence indicates that TMR is effective at modulating a variety of memory traces. While the unique effects of TMR protocols make it difficult to fit it cleanly within any single category discussed here, it is clearly another exciting new tool available to sleep researchers that may aid in the continued dissection of sleep’s effect on emotional memory processing.

### Sleep during extended consolidation

Following the early consolidation phase, emotional memories enter into a period of extended consolidation. This stage in memory formation refers to any additional consolidation processes that occur to a memory after it transitions from its labile state during the early consolidation phase into a more stable memory trace and extends all the way until the memory is retrieved, reactivated, or forgotten. With regard to emotional memory, this period remains one of the least studied involving sleep manipulations, though memory studies using non-emotional tasks indicate that sleep may expedite the transition of memories from the early to extended consolidation phase (Walker et al., 2002, 2003; Stickgold, 2005) and that interference training or manipulating sleep after a memory enters this phase of memory formation appears to have little influence on later retrieval (Stickgold et al., 2000; Walker et al., 2003; Ellenbogen et al., 2006; Alger et al., 2012). Still, an important question continues to be in the absence of sleep, at what point (or does) the waking brain transition a memory from the early to extended consolidation phase so that it might be less influenced by other processes such as sleep and stress.

While substantial research has aimed at the early consolidation stage of emotional memory processing, very few studies have explored this extended consolidation period (see Table 2), and, to our knowledge, only three studies have utilized protocols meant to specifically target this stage. In a final sample of 265 Dutch children, Vermeulen et al. (2017) had participants encode 30-word pairs (10 positive, 10 negative, 10 neutral) at 7am or 7pm, followed by an immediate recall test. Participants then either had a 12-h delay of daytime wakefulness or nocturnal sleep (targeting early consolidation) or a 24-h delay in which sleep occurred either shortly after encoding (7 pm condition) or after a normal day spent awake (7 am condition), targeting the extended consolidation period. They found that while more arousing words did result in better immediate and delayed recall, there was no main effect or interaction of sleep for any of the conditions. They also reported no deterioration in memory from immediate to delayed recall across both the 12-h and 24-h periods, regardless of sleep placement (Vermeulen et al., 2017). Another study utilized essentially the same sleep manipulation protocol (12-h delay of sleep vs. wake and 24 h delay of sleep-first vs. wake-first), but used the emotional memory trade-off paradigm in young adults (Payne et al., 2012). As noted above, the sleep effect was strongly diminished in the wake-first group compared to the sleep-first condition, suggesting that sleep may have less of an impact on emotional memory processing once the memory reaches this stage, at least for some paradigms. A final study recently utilized a similar sleep-first vs. wake-first protocol, but memory was tested for negative and neutral scenes on three separate occasions across the protocol: immediately following encoding, 12-h after encoding, and 24-h after encoding (Carollo et al., 2022). While immediate testing showed no differences, at the 12-h mark memory was clearly benefited by a sleep period, though this effect was generalized and not limited to negative pictures. Interestingly, testing after the 24-h delay showed that the sleep-first group showed deterioration in memory in the subsequent 12-h of wakefulness, while memory was largely preserved from the 12- to 24-h test in the wake-first group after receiving their opportunity for sleep in the second half of the protocol (Carollo et al., 2022). Importantly, while the authors clearly state that the intention was to assess delayed sleep-dependent consolidation on emotional memory, it is likely that the 12-h testing session reactivated memory traces for the stimuli that had been set aside for the 24-h assessment. Thus, in this design elements of reactivation and reconsolidation may have been in effect as well.

**TABLE 2:** Studies aimed at investigating the effect of sleep on the extended consolidation phase of emotional memory processing.

Study	Human subjects (Y/N)	Genders	Age (M ± SD)	N	Between-groups/Within-groups	Sleep manipulation	Stimulus material	Emotions	Immediate test (Y/N)	Time of day control (Y/N)	Main effect of sleep (effect size)	Interaction sleep × emotion (effect size)
Atienza and Cantero, 2008	Y	Mixed	21 ± NR years	28	Between	TSD/S	Pictures	NNP	N	N	Y (NR)	N (NR)
Carollo et al., 2022	Y	Mixed	23.6 ± 3.2 years	40	Between	SF/WF	Pictures	NN	Y	N	Y*(η_*p*_2 = 0.10)	N (η_*p*_2 < 0.01)
Payne et al., 2012	Y	NR	18-25 years (M ± SD: NR)	71	Between	SF/WF	Pictures (ETO)	NN	N	N	NR	Y (NR), negative objects better remembered in sleep-first compared to wake-first conditions indicating larger influence in early compared to extended consolidation
Sterpenich et al., 2007	Y	Mixed	22.3 ± 2.7 years	40	Within	TSD/S	Pictures	NNP	N	N	Y (NR)	N (NR)
Sterpenich et al., 2009	Y	Mixed	22.3 ± 2.7 years	40	Within	TSD/S	Pictures	NNP	N	N	N (NR)	N (NR)
Vermeulen et al., 2017	Y	Mixed	10.5 ± 0.8 years	265	Between	SF/WF	Words	NNP	Y	N	N (OR = 1.06)	N (OR = 1.10)

Style of Tables adapted from Davidson et al. (2021). Table includes studies included as part of this narrative review, and is not necessarily a comprehensive list of all published works. Y, yes; N, no; Mixed, not restricted to a single reported gender; ETO, emotional trade-off task; SF/WF, 24 h protocol with sleep-first vs. wake-first groups; TSD/S, total sleep deprivation vs. sleep; NN, negative and neutral; NNP, negative, neutral, and positive; NR, not reported.

*For “hit rate” but not d-prime calculation of memory performance.

Given the uncertainty surrounding the timing at which memories may transition from the early to extended consolidation stage in the absence of sleep, there are two additional studies that may have some relevance in this investigation. Atienza and Cantero (2008) had participants encode negative, neutral, and positive pictures either at 11 am or 5 pm prior to a normal night of sleep or TSD. Participants returned a week later for a recognition test using a Remember/Know memory assessment (Tulving, 1985). They found a main effect of sleep on Remember judgments such that TSD participants had poorer overall recall. They also report that while Remember judgments for neutral images were more impaired by sleep deprivation (41%) compared to emotional information, the interaction between sleep and emotion was not significant (Atienza and Cantero, 2008). While additional information might be able to be gleaned from the different consolidation periods prior to the first bout of sleep or sleep deprivation, the authors used the different encoding times as a means to take circadian influences into consideration and collapsed the groups for analysis. Similarly, Sterpenich et al. (2007, 2009) had participants encode negative, neutral, and positive pictures between 3:30 pm and 8:30 pm before sleep or TSD, and participants were allowed two nights and 6 months of recovery sleep prior to testing. Memory was poorer overall for TSD participants at both testing sessions. After the three-day delay, “Remember” judgments specifically deteriorated for neutral and positive images while memory for negative images was not impacted by lack of sleep, though this effect was not present at the 6 months delay, possibly due to high variability in memory capacity at that time (Sterpenich et al., 2007, 2009). Interestingly, less vividly retained memories as indicated by “Know” judgments did not show as strong of a sleep effect even after the short delay (see section “Discussion”). Of relevance to this review, however, while the authors did isolate the consolidation period in their design, the differential consolidation periods prior to the first bout of sleep or sleep deprivation were not a factor in their analysis.

**FIGURE 5:**
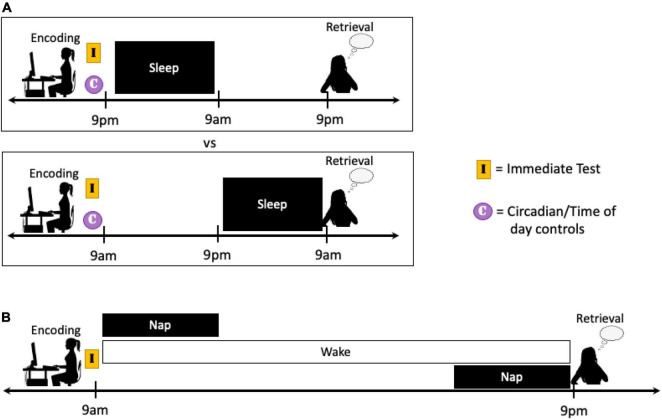
Schematics of two potential study protocols designed to target the effects of sleep manipulation on the extended consolidation period. **(A)** Similar to the studies described in the text, longer protocols can be implemented so the sleep manipulation occurs after either a short or extended delay following encoding. In cases where encoding takes place at different times of day, immediate testing and/or circadian controls should be employed when possible to account for differences in sleep pressure and circadian phase. **(B)** Nap studies could also be utilized to better distinguish the point at which a memory transitions from the early to the extended consolidation stage. In these designs, a nap can occur either shortly following encoding or after an extended delay and performance can be compared to participants that remain awake the entire consolidation period. Modulating the placement of the extended nap in a systemic fashion could help determine when this transition occurs in the waking brain, or if sleep is necessary.

Future studies that aim to differentiate the effects of full nights of sleep on the early and extended consolidation periods of emotional memory would benefit from the use of 24- or 48-h study designs. In these studies, there is at least a 24-h delay between the encoding and retrieval of emotional information, and sleep or sleep deprivation is scheduled to occur either shortly after encoding or after an extended delay. In cases where the encoding sessions take place at different times of day (e.g., 9am and 9pm), it is critical to attempt to control for differences in circadian rhythm, time of day effects, and homeostatic sleep pressure using immediate tests and/or circadian control groups (Figure 5A). Nap protocols may also be used to gain better understanding of the amount of time that needs to pass before the early consolidation period transitions into the extended consolidation period. In such a design, comparisons can be made between participants that (1) nap shortly after encoding, (2) nap at variable, substantial delays after encoding, and (3) remain awake until testing. This design would have the added benefit of keeping circadian and time of day influences consistent between groups (Figure 5B).

### Sleep prior to retrieval

Memory retrieval refers to the process of activating the neural memory trace of our previous experiences to call to mind a specific item or event or to perform a specific task. With regard to declarative emotional memory, it is at this phase of memory that enhancements in memory for emotional vs. neutral information following sleep or wake are tested. Importantly, retrieval of emotional memory has been tested in a variety of different ways (e.g., free recall, cued recall, recognition, forced choice, etc.), and recent evidence has indicated that certain tasks and types of testing (e.g., free recall, emotional memory trade-off) may be more sensitive to picking up on selective benefits of sleep for emotional content (Lipinska et al., 2019; Schäfer et al., 2020; Davidson et al., 2021). Additionally, for some tasks (including emotional memory) the effects of sleep may be more pronounced when examining changes in pre-sleep to post-sleep testing rather than comparing memory performance following the sleep manipulation alone (Lipinska et al., 2019; Schäfer et al., 2020).

To our knowledge, no human studies have *specifically* targeted the influence of sleep on this pre-retrieval stage of emotional declarative memory processing. This paucity of data is quite surprising given relatively robust animal models that not only indicate that sleep loss prior to retrieval of emotionally laden information most certainly does impair memory performance, but there might be sex differences in the degree of impact as well (Fernandes-Santos et al., 2012), and also given recent models of emotional memory that emphasize effects operating at retrieval (e.g., Bowen et al., 2018; Talmi et al., 2019). As such, there is an obvious need for human research that intentionally utilizes sleep manipulations to specifically target the retrieval stage of emotional memory processing.

**FIGURE 6:**
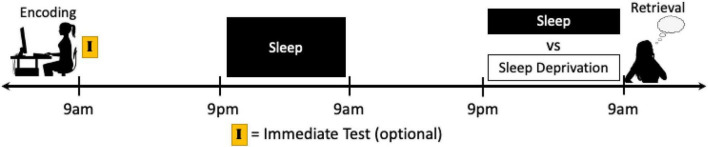
Schematics of a potential study protocol designed to isolate the influence of sleep just prior to retrieval on emotional memory performance. Participants can complete an encoding task and after a night, or multiple nights, of sleep to ensure that memories have transitioned to the extended consolidation stage, sleep can be manipulated just prior to retrieval. In this way, sleep vs. sleep loss can specifically target the retrieval of emotional memories while also controlling for circadian rhythm and time of day effects. Moreover, an immediate test directly after encoding can be used to determine within-subject memory changes due to the different conditions, however it must be kept in mind that the testing of memory itself may influence subsequent memory performance (see Footnote 4 and section “Discussion”).

This lack of targeted research is in sharp contrast with the fact that a majority of sleep and emotional memory studies do ultimately manipulate sleep prior to retrieval by shifting the occurrence of sleep in relation to learning. Thus, as part of this manipulation, the proximity of sleep to retrieval has also been shifted (i.e., when sleep is shifted further away from encoding, it is also shifted closer to retrieval), but the results are not discussed as such. For instance, in typical nocturnal sleep vs. daytime wakefulness and nap vs. no nap study designs, when memory is tested immediately following the sleep manipulation there is a difference in homeostatic sleep pressure during retrieval. Additionally, in sleep vs. sleep deprivation or sleep restriction protocols, if recovery sleep is not permitted prior to the memory assessment then sleep loss is having an active effect on retrieval processes as well. Some of these influences can again be accounted for via immediate tests and circadian control groups. To specifically target this phase in memory processing, however, longer study protocols would need to be employed. For instance, participants could all complete the encoding session at the same time of day on Day 1, and then return several days later, after the memory has reached the extended consolidation phase, to have their memory tested directly following a night of sleep deprivation or sleep or directly following a nap or no nap (Figure 6). Such a protocol would also benefit by keeping the encoding and testing sessions at the same time of day for both conditions, thereby eliminating time of day and circadian effects.

### Sleep post-retrieval/during reconsolidation

The evolution of a memory is not complete after retrieval. Instead, once a memory is retrieved or reactivated (i.e., a brief reminder of the memory content), it has been shown to return to a labile state that again makes it more prone to influence, modulation, and forgetting^6^ (Stickgold and Walker, 2007; Tronson and Taylor, 2007; Nader and Einarsson, 2010; Alberini and LeDoux, 2013; Agren, 2014; Cassini and Lee, 2018; Simon et al., 2020), much like the period of early consolidation. Several studies have suggested that sleep plays an important role in re-stabilizing declarative episodic memories following reactivation (Klinzing et al., 2016; Moyano et al., 2019; Bryant et al., 2020). For instance, in one study that used retrieval-induced forgetting as a way to ascertain whether a memory was in a labile state (Moyano et al., 2019), results led the authors to propose that 90 min of sleep may be sufficient to accelerate memory re-stabilization, shortening the time over which memories are susceptible to interference. Joensen et al. (2022) recently reported that targeted memory reactivation during sleep can lead to retrieval induced forgetting – but not for memories that had been reactivated just prior to sleep, perhaps consistent with other evidence (Klinzing et al., 2016) that a brief sleep epoch can specifically improve retention of recently reactivated memories.

To our knowledge, only a single study has directly examined the effect that sleep may have on the reconsolidation of emotional episodic memories (see Table 3). Azza et al. (2022) asked participants to undergo an “imagery rescripting” manipulation in which they retrieved an autobiographical memory and reinterpreted it so as to make it less aversive. After this retrieval of the memory, half of the participants were given a nap opportunity while the others stayed awake. 90 min later, the nap group showed a reduced heart rate response to the negative memory script as compared to the wake group, and spindle density was correlated with this reduced arousal response. The authors argued that their results provided the first evidence of a beneficial role for sleep in the adaptive reconsolidation of aversive memories, though the effect did not persist after a 1 week delay (Azza et al., 2022).

**TABLE 3:** Studies aimed at investigating the effect of sleep on the post-retrieval/reconsolidation phase of emotional memory processing.

Study	Human subjects (Y/N)	Genders	Age (M ± SD)	N	Between-groups/Within-groups	Sleep manipulation	Stimulus material	Emotions	Immediate test (Y/N)	Time of day control (Y/N)	Main effect of sleep (effect size)	Interaction sleep × emotion (effect size)
Azza et al., 2022	Y	Mixed	23.2 ± 3.3 years	44	Between	Nap	Autobiographical Memory*	NN	N	N	NR	Y (η_*p*_2 = 0.12)
Gui et al., 2019 **	Y	Mixed	Young: 18–25 Older: 58–78 (M ± SD: NR)	119	Between	DW/NS	Pictures	NNP	Y	N	Y (η2 = 0.07)	N (η2 = 0.02)


Style of Tables adapted from Davidson et al. (2021). Table includes studies included as part of this narrative review, and is not necessarily a comprehensive list of all published works. Y, yes; N, no; Mixed, not restricted to a single reported gender; DW/NS, daytime wake vs. nocturnal sleep; Nap, daytime nap vs. no nap protocol; NN, negative and neutral; NNP, negative, neutral, and positive; NR, not reported.

*Memory was tested as heart rate reactivity to rescripted negative autobiographical memory after nap manipulation. Effect did not persist at retests 1 week later.

**Only reporting results from the second memory task after reactivation during the first memory task.

While this is the only study to directly examine the reconsolidation phase for emotional memories, there is other suggestive evidence that sleep may affect the way that emotional memories are reconsolidated after initial retrieval. In particular, the effects of sleep on emotional memory can unfold over time and across retrievals – a pattern broadly consistent with the proposal that sleep is affecting the way that memories are reconsolidated after retrieval. For instance, upon a short-delay retrieval task, sleep primarily boosted young adults’ neutral memory, whereas after a longer delay, benefits were revealed for young adults’ positive memory as well (Gui et al., 2019).

**FIGURE 7:**
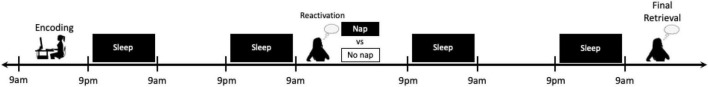
Schematics of a potential study protocol designed to target the effects of sleep manipulation on post-retrieval, reconsolidation processing of emotional memory. In the typical reconsolidation study design, the encoding, reactivation, and retrieval sessions typically take place days apart with full nights of sleep in between, allowing for the investigation into the specific effects of sleep manipulation after reactivation using nap or sleep deprivation protocols. While sleep has been shown to be an important component of the reconsolidation process in other forms of declarative memory, investigations into the effects on emotional episodic memory remain scarce.

Fortunately, the gold-standard study protocols designed to target the effects of sleep during this post-retrieval, reconsolidation period are typically proficient at isolating the effect of sleep specifically on this phase of memory processing. In many of these studies, the encoding session, the reactivation manipulation (e.g., reactivation vs. no reactivation), and the retrieval session each take place days apart, with sleep being manipulated immediately following reactivation (e.g., nap vs. no nap or sleep vs. sleep deprivation protocols; Figure 7). By providing multiple nights between each study session, it can be assured that the memories have entered (or re-entered) the extended consolidation phase before manipulation or testing and that the effects of the sleep manipulation are no longer active at the time of retrieval. Additionally, the encoding task, reactivation task, and retrieval task can all be scheduled at the same time of day between conditions, minimizing time of day and circadian effects and controlling homeostatic sleep pressure at the time of each assessment. As noted, an important future direction will be to intentionally add emotional elements into these designs to differentiate the impact of sleep on the reconsolidation of emotional and neutral aspects of memory. However, a number of studies to date that have conducted multiple memory tests on information encoded in a single session may have unintentionally or inexplicitly included reconsolidation into their study design. For instance, in studies in which emotional and neutral pictures and words are encoded and memory for half of the material is tested 12 h later and memory for the other half of the stimuli is tested days or weeks later, it is possible that the material not presented during the initial 12 h test was reactivated and reconsolidated prior to the final test session.

## Discussion

The primary motivation for this review was the recent surge in comprehensive publications describing the rise in mixed findings with regard to sleep and emotional episodic memory processing (Lipinska et al., 2019; Schäfer et al., 2020; Davidson et al., 2021). The long-standing belief that sleep interacts with emotionally arousing information to prioritize it in long-term declarative memory over neutral information has been called into question after it was determined that a *majority* of investigations were unable to replicate this effect. As it stands, it is important to reassess the current literature with regard to different factors that may be responsible for generating these mixed findings (e.g., type of stimuli and testing, intensity of stimuli, amount of time between encoding and retrieval, the type of sleep manipulation, etc.). Here, we contribute to this work by reviewing the literature in the context of the placement of sleep with regard to the formation and evolution of an emotional memory.

In general, the extreme absence of sleep through manipulations such as TSD appears to have a near ubiquitous negative effect on overall memory performance compared to normal sleeping behavior, regardless of what memory stage it is introduced (Drummond et al., 2000; Stickgold et al., 2000; Walker and Stickgold, 2006; Sterpenich et al., 2007; Wagner et al., 2007; Fernandes-Santos et al., 2012; Tempesta et al., 2016, 2017). Moreover, a period of sleep frequently has a beneficial effect on overall memory as compared to a matched period of daytime wakefulness (e.g., Prehn-Kristensen et al., 2009; Baran et al., 2012; Alger and Payne, 2016; Lehmann et al., 2016b; Whitehurst et al., 2016; Bolinger et al., 2018), though this is not always the case (e.g., Ashton et al., 2019; Bolinger et al., 2019; Huan et al., 2020). With regard to sleep prior to encoding, TSD seems to impair memory for all valences (Kaida et al., 2015), though may have the *least* detrimental effect on subsequent memory for aversive stimuli (Walker and Stickgold, 2006; Tempesta et al., 2016) resulting in what may appear to be a prioritization of negative information. As previously reviewed (Lipinska et al., 2019; Schäfer et al., 2020; Davidson et al., 2021), the literature on the effects of sleep on the early consolidation period of emotional memory processing is quite mixed. While a majority of studies indicate that sleep does have a positive effect on overall memory, regardless of valence, only a small portion of studies indicate that it specifically prioritizes emotionally arousing information during this period (Davidson et al., 2021). The exception to this general trend has been studies that use the emotional memory trade-off paradigm (Kensinger et al., 2007), which has indicated that sleep during the early consolidation period reliably leads to enhanced memory for negative central details over neutral peripheral details (Payne et al., 2008, 2012, 2015; Payne and Kensinger, 2011; Alger et al., 2018).

Studies specifically targeting the effects of sleep and sleep loss on emotional episodic memory during the extended consolidation, pre-retrieval, and post-retrieval stages of memory processing remain particularly scarce. For extended consolidation, one study of word pairs in children found no sleep effects on memory regardless of whether sleep occurred during early or extended consolidation (Vermeulen et al., 2017), while a study using the emotional trade-off paradigm found that delaying sleep to the extended consolidation period resulted in less of an impact than sleep obtained during the early consolidation period (Payne et al., 2012). Finally, the sole study that investigated the impact of sleep on the reconsolidation of emotional episodic memories found that participants that napped after imagery rescripting ended up with a reduced heart rate response to the negative memory script the next time it was presented (Azza et al., 2022). Notably, most of the work cited here was not able to completely eliminate all potential overlap between sleep stages and confounding circadian and sleep pressure effects, motivating future work that continues to work toward isolating the effects of sleep on each stage of emotional memory processing.

### Targets for future research

Given the lack of research in some of these windows of episodic emotional memory development, we also aim to support future research by identifying the least studied memory stages with regard to the effects of sleep and emotional declarative memory processing and seek to re-initiate a discussion on the best protocol practices to attempt to target each memory phase with minimal overlap with other phases. The vast majority of research has been on the effects of sleep during the early consolidation phase. One possible explanation for the hyper-focus on this stage of emotional memory processing with regard to sleep is that much of the foundational research has indicated that sleep (Stickgold et al., 2000; Walker et al., 2003; Stickgold, 2005) and emotion (McGaugh, 2000, 2004) each individually have major influences on memory during this period, making it a prime target when searching for the interaction between these two factors. Given the increase in mixed findings of this effect, however, it would be worthwhile to broaden the search and isolate the impact of sleep manipulations on other, less studied stages of emotional episodic memory development.

The extended consolidation and post-retrieval periods currently remain two of the least studied periods of emotional memory processing. Further, while sleep is manipulated prior to retrieval in many commonly used study designs, especially when the effects of Process S and Process C are taken into consideration, essentially no human research has been done with the explicit goal of targeting this phase of emotional episodic memory processing. This is rather surprising given that it is a period that can be relatively well isolated from the other phases of memory processing, animal models suggest that the absence of sleep pre-retrieval impacts memory performance, and recent models of emotional memory that emphasize effects operating at retrieval (e.g., Bowen et al., 2018; Talmi et al., 2019). Thus, while the vast majority of sleep and emotional memory research has targeted the early consolidation phase of memory processing, recent reviews indicate that the findings have been quite mixed with small effects at best. By broadening the scope of the different ways that sleep can influence an emotional memory and researching each phase in a deliberate, stepwise manner, we may identify confounding influences leading to this surge in mixed results and better understand the strengths and limitations of this effect.

### Need for novel protocol development

Research in this area would also benefit from increased ingenuity with regard to protocol development. As discussed above, many of the traditional sleep vs. wake, sleep vs. sleep deprivation, and nap vs. no nap study designs manipulate sleep and wake at multiple phases of memory processing, especially when Process S and Process C are taken into consideration. Additionally, several studies in this area have used “early sleep vs. late sleep” paradigms to try to target differential effects of slow wave sleep and REM sleep on emotional memory processing (Wagner, 2001; Wagner et al., 2006; Groch et al., 2013, 2015; Sopp et al., 2017). While these studies do successfully manipulate the makeup of the sleep macroarchitecture during the early consolidation period, there are also group differences in sleep homeostasis and circadian effects during encoding and retrieval in action as well. This overlap in memory phases may be particularly confounding when considering emotional memory as any differential processing between emotional declarative memory and other forms of declarative memory is predicated on the fact that the experience and processing of an emotionally arousing event triggers unique neurophysiological responses that tag that information as being particularly relevant and worth prioritization in our memory (Payne and Kensinger, 2018; Payne, 2020). As such, any change along the pipeline of emotional memory processing may have substantial impact on how it is ultimately remembered and retrieved, and factors like sleep pressure and circadian phase likely interact with the initial experience and early consolidation of an emotional event.

We have highlighted some potential protocol designs above aimed at better dissociating the different memory phases from one another, but even several of these fail to completely control for all potential sleep-related effects and overlap between memory phases. Beyond our suggestions, there are also substantial opportunities – and frankly a need – for collaboration between sleep and circadian researchers. For instance, while circadian control groups may aid in statistically controlling for circadian effects, a forced desynchrony experiment could systematically disentangle the contributions of sleep from circadian rhythm at a neurophysiological level (Dijk et al., 1992, 1999; Zhou et al., 2011). In these protocols, participants are scheduled to live on days that are substantially shorter or longer than the typical, free-running 24.1–24.2 h circadian period (Borbély and Achermann, 1999). Sleep episodes are thereby scheduled at different periods within the circadian phase. In this way, the effects of sleep and circadian phase can be disassociated and compared. Not only has this protocol been used to isolate EEG components driven by Process S and Process C (Dijk and Czeisler, 1995), but it has also already been used to distinguish differential effects of homeostatic sleep pressure and circadian phase on non-emotional cognitive tasks involving planning and sequence learning (Dijk et al., 1992; Cajochen et al., 2004). While herculean in nature, it is this type of elegant study design that is going to ultimately allow us to distinguish the effects between Process S and C on emotional memory consolidation with certainty, and by piggybacking on a study by circadian sleep researchers, both the cost and effort of the memory study can become minimal.

While our goal here is to encourage the field to move further with collaboration and study design innovation, we also acknowledge that several of the protocols that we have proposed suggest taking a study design that can typically occur in a single visit (e.g., encoding and testing immediately pre- and post-sleep or sleep deprivation) and stretches it out across multiple visits. These additions would increase logistical complexity, could reduce the range of participants able to participate, and cost and may become prohibitive. Given these barriers, we are not suggesting that there is no knowledge to be gained from the standard study designs. However, the limitations of these designs should be openly discussed and understood, and ideally immediate testing and/or circadian controls can be employed to help account for these additional influences when longer protocols are not possible. Ultimately, our goal is to promote increased deliberateness with which we conduct our studies to intentionally isolate the memory phase that we are studying and acknowledge the potential influence of overlap between phases when that is not possible.

### Practical considerations

As part of this call for protocol development, there are several open questions that merit further investigation of their own. To begin, there are a number of practical elements that would benefit from heightened consideration in this line of research. For instance, it is important to understand how sleep placement might interact with some of the other elements that have been identified as possibly important for emotional memory processing, such as stimulus intensity and the type of encoding and retrieval assessment used. One challenge faced by researchers is the substantial diversity in tasks and assessments used to assess episodic emotional memory, which makes it difficult to track trends across the literature. The lack of standardization in the field is likely due to the fact that both episodic memory and the human experience of emotion are sufficiently complex that there can be no single task that captures all of their canonical features and interactions. As such, a variety of tasks have been developed in an attempt to capture the different components of emotional memory that can be reasonably measured within a laboratory space. While this does inevitably lead to a ‘weakness,’ in that there are a number of mixed findings that must be sorted out to try to make sense of the literature, it is also a potential ‘strength’ in that when results do begin to consistently coalesce around a common effect, our confidence that the effect is legitimate increases, especially when there is slight variability in tasks and stimuli used.

The field of sleep and emotional memory is currently at a crossroads as a growing number of studies fail to replicate the preferential retention of emotional memory elements associated with sleep. Similar to our focus on the “placement of sleep” here, we are in need of additional task-centric conversations in order to make better sense of the field as it stands and to help guide future research. To begin, it will be imperative that effect sizes are reported in this line of research. Comparing the magnitude of effect for different emotional memory probes will be invaluable in disentangling where the effects of sleep are consistent. With the normalization of this level of data transparency moving forward, similar reviews and meta-analyses can be conducted at regular intervals with a focus on how task designs, stimuli, retrieval probes, and calculations of memory relate to the effect of sleep, an effort that has already been initiated by some of our colleagues (e.g., Lipinska et al., 2019; Schäfer et al., 2020; Davidson et al., 2021).

An example of task type interacting with the effect of sleep on emotional memory consolidation is the relatively consistent success of studies finding a sleep effect when utilizing the emotional memory trade-off (ETO) paradigm (Kensinger et al., 2007; Payne et al., 2008). One element of the memory trade-off design that may be essential for revealing sleep x emotion interactions on memory is that encoding is *incidental*, and even in cases where participants might suspect a memory test, no participant reports that they expected their memory to be tested for *components* of scenes. This design may therefore mirror some of the most important aspects of real-world episodic memories: Events are multifaceted, we tend to be processing those events for reasons other than memorizing what is happening to us, and we are almost never re-presented with an entire event (a standard old/new recognition test) nor asked to recall with no cues everything that we experienced (a standard recall test). The incidental nature of the memory task may be particularly important, as a study by Bennion et al. (2016) found that when trade-off stimuli were presented under conditions in which people either knew that their memory would be tested or were unaware of the subsequent memory test, sleep prioritized emotional vs. neutral content when studied in the incidental condition, but not when studied in the intentional encoding condition. Another benefit of the ETO task is that some versions assess both strict veridical memory and more lenient general or gist memory. Importantly, assessing specific, veridical memory for previously viewed stimuli is just one way that memory can be assessed, and the ETO paradigm is just one example of a task that assesses memory at different levels of specificity. A number of the studies highlighted above distinguish between the strength (e.g., Remember vs. Know paradigms; Sterpenich et al., 2007, 2009; Atienza and Cantero, 2008) or resolution of memory (e.g., veridical vs. gist memory in ETO task or Deese–Roediger–McDermott task; McKeon et al., 2012; Payne et al., 2012; Kurz et al., 2019), which may be more ecologically similar to how emotional events are actually retained in our memory. Both the type of probe and the strength of the memory trace may be important elements that interact with the placement of sleep leading to more or less reliable findings and warrant further investigation and subsequent reviews aimed specifically at their contributions to this relationship. Ultimately, there may be a certain set of factors that generate the most reliable effects of sleep on emotional memory processing.

Another practical question is the effect of multiple memory tests in longer sleep and memory protocols. For example, some studies have had participants test on half of the material that they encoded after a 12-h period of sleep or wake, and then return to the lab several days or a week later for a second memory test on the other half of the materials. These memory tests are frequently viewed as being equivalent in nature, just over longer delay intervals. Critically, however, many memory reconsolidation periods can use a similar protocol in which memory for some of the items or just the task itself may be activated, followed by another delay before final testing. In studies that utilize these multiple tests, it is important to understand how an initial memory test (and whether it is followed by sleep or wakefulness) might reactivate and reconsolidate the material that has yet to be tested.

A related point is that cognitive models of memory are increasingly recognizing the importance of context effects at retrieval in predicting what information comes to mind and what information is not able to be accessed. Context can refer to the match between the encoded context and the retrieved context or to fluctuations in context across the retrieval epoch that may be related to the earlier information that has been brought to mind (see Bowen et al., 2018; Talmi et al., 2019). As we noted earlier, many sleep-wake designs that attribute their effects to influences on consolidation are, in fact, also manipulating sleep pre-retrieval. Future work will be well served to consider how this may affect results. For instance, if sleep enhances consolidation, this benefit could be masked when those in the sleep condition are experiencing a change in context from pre-encoding (no sleep) to pre-retrieval (sleep) while those in the wake condition are not.

Beyond these considerations that relate to the placement of sleep within the context of the memory experiment, there is the challenge of considering sleep history and individual differences as well. Pre-encoding sleep could be thought to include the aggregation of multiple nights of sleep or sleep loss leading up to the experience of an emotional event, and it is likely that all stages of emotional memory processing are affected in those that have consistent difficulty sleeping. Moreover, research has indicated that individual differences (Chuah et al., 2009) and demographic factors such as sex (Killgore et al., 2008b; Fernandes-Santos et al., 2012; Goldstein et al., 2013) and age (Philip et al., 2004; Duffy et al., 2009; Talbot et al., 2010) may also impact the degree to which we are affected by sleep loss. While tools such as actigraphy and sleep logs can give us a peek into the typical sleeping habits of our participants, it is likely that more than a single night of sleep influences our interpretation of and interaction with emotional events. More research into these individual differences will contribute to our broader understanding of the effects of sleep on emotional perception and memory processing.

### Theoretical considerations

Beyond these practical considerations, there are a number of broader, theoretical questions that need to be addressed as well. For instance, one consideration particularly relevant to this review is that “sleep’s effect” on emotional memory may highly depend on the cognitive and affective processing that is engaged surrounding the placement of the sleep manipulation. As noted, a majority of sleep and emotional memory research has focused on the early consolidation stage of memory processing. This stage has been particularly tantalizing for researchers given evidence that suggests distinct neurophysiological responses rise during the experience of arousing events that tag that experience for prioritized processing during the early consolidation period, thereby enhancing long-term retention (Payne and Kensinger, 2018; Payne, 2020). However, isolating the effects of the sleep manipulation on the different stages of memory processing could reveal other co-occurring mechanisms that add to the overall variability of the sleep effect. For instance, perhaps the most established impact of sleep deprivation is its negative effect on attentional resources (Durmer and Dinges, 2005). If the effect of a sleep manipulation could be isolated to the pre-encoding stage of memory processing, we may find that a lack of attentional resources leads to an increased focus on emotional information (i.e., emotional content grabs our attention even when tired). Thus, the prioritization of this information being preserved long-term may begin even before any preferential processing can occur during early consolidation. Taking a step-wise approach to investigating the effects of different placements of sleep manipulations across the stages of emotional memory processing identified here would help us to better understand the different effects that contribute to the impact of sleep on emotional memory and their magnitude.

Another factor that remains unclear is how long it takes for a memory of any kind, let alone an emotional memory, to transition from the “early” to “extended” consolidation period. Indeed, many things about the time course of a memory that might have seemed to be settled science – such as the exponential forgetting curve discovered by Ebbinghaus – have recently been drawn into question (Radvansky et al., 2022). It seems as though sleep itself expedites this transition into “extended” consolidation, but it remains to be determined if the waking brain can make this transition alone, and if so, how long it takes. If the waking brain can make this transition alone, then the question remains about the differential impact of manipulating sleep at these different periods.

More generally, most frameworks for understanding the effects of sleep and emotional memory consolidation seem to build from models that assume a similar hippocampal-dependency for emotional and neutral memory consolidation, perhaps motivated by models of emotional memory that focused on synergistic relations between the amygdala and hippocampus (e.g., McGaugh, 2000, 2004). Yet other models of emotional memory propose diminished roles for hippocampal-dependent processes, and increased roles for amygdala-dependent processes, in long-term retention of emotional components of episodic experiences (e.g., Yonelinas and Ritchey, 2015; Bisby et al., 2016). Framing effects of sleep within the context of these models may lead to different predictions for how sleep influences emotional memory vs. neutral memory consolidation.

### Beyond emotional memory

Finally, we want to reiterate that here we solely focused on the relationship between sleep placement and declarative memory for the content of emotional experiences. One of the major challenges in the human sleep and memory field in general is that there is extensive variability in the research that may have substantial impact on the results that continues to be poorly understood. These include factors such as the placement and type of the sleep manipulation, the type of task or memory test used, and the type and intensity of the emotional stimuli. As such, doing similar breakdowns of different types of memories (e.g., working memory, procedural memory, fear memory, autobiographical memory, etc.) would be a worthwhile endeavor that would ultimately lead to a greater understanding of the strength and limitations of the effect of sleep on overall memory processing. Additionally, this review exclusively focused on sleep’s role in memory retention for the content of emotional experience. Substantial research has indicated that sleep may also play an active role in modulating the emotional tone or affectivity associated with emotional experiences (Walker and van Der Helm, 2009; van der Helm et al., 2011; Baran et al., 2012; Cunningham et al., 2014a; Bolinger et al., 2019). As such, the relationship between sleep and affective modulation of emotional experiences would benefit from a similar discussion on the effects of sleep placement, especially given the additional clinical implications of this effect.

## Conclusion

In summary, initial evidence suggests that sleep and sleep loss has differential effects depending on what phase in the evolution of an emotional memory it is manipulated. Here, we operationalized these stages as (1) prior to encoding, (2) immediately following encoding during early consolidation, (3) during extended consolidation away from initial learning, (4) just prior to retrieval, and (5) post-retrieval/reconsolidation. While some of these phases have been extensively studied with regard to the impact of sleep (i.e., sleep during early consolidation), others remain under-researched, particularly in the context of intentional research targeting that phase of emotional memory processing (i.e., extended consolidation, prior to retrieval, reconsolidation). Moreover, the absence or presence of sleep is often manipulated in multiple phases of emotional memory processing in a number of the typical study designs used in the field, with factors such as sleep pressure, time of day, and circadian effects not always taken into consideration. Given the recent surge of mixed findings with regard to the role of sleep in emotional memory processing, future research should aim to disentangle the effects of sleep on these different components in the evolution of an emotional memory, and memory in general, through intentional, targeted study protocols that do their best to account for sleep homeostatic and circadian influences.

## Author contributions

TC was primary author responsible for drafting initial manuscript with EK and RS providing additional comments, edits, feedback, material, and direction. All authors contributed to the concept and major elements of the review.

## Footnotes

1 Of course, manipulations of sleep pre-encoding can also be considered to affect the encoding phase itself (i.e., someone who is sleep-deprived pre-encoding enters the encoding phase in that state).

2 As with pre-encoding manipulations, sleep manipulations that occur just prior to retrieval will necessarily affect the retrieval phase itself.

3 Critically, in most cases there is no “perfect” study design in which all sleep and circadian influences are entirely limited to a single stage of emotional memory processing. Our goal here is to motivate future work that makes intentional effort either to minimize the overlap between stages or to embed conditions that control for differences in stages that are not the intended target, with the ultimate goal of better understanding some of the influences that might account for the mixed findings in the field.

4 While this design fixes some problems, it may introduce others. First, this design requires the use of “intentional encoding” designs, whereby participants know their memory will be tested; otherwise, the comparison of the short- and long-delay performance would be like comparing apples (a surprise short-delay memory task) and oranges (an expected long-delay memory task). Second, asking participants to retrieve a subset of items on a short-delay task may initiate other phenomena (e.g., retrieval-induced forgetting effects), which could in turn affect performance on the long-delay task.

5 While sleep was manipulated pre-encoding, in this design, TSD would also affect the retrieval of material on the short-delay test. Thus, the Condition × Design interaction could be considered a comparison of the effect of TSD on retrieval (TSD would affect short-delay but not long-delay retrieval).

6 By “forgetting,” we refer to the failure to retrieve a previously accessible memory (e.g., if you remember what you ate for breakfast yesterday morning but can no longer recall that information 2 weeks from now, that would be an instance of forgetting). Recent evidence suggests that this may occur via either passive or active processes and likely involves the deterioration of molecular and cellular memory traces or erosion of the memory cell circuit that would make it unresponsive to recall mechanisms (Davis and Zhong, 2017; Anderson and Hulbert, 2021).
